# Supplementary material for: Cost-effectiveness of a preferred intensity exercise programme for young people with depression compared with treatment as usual: an economic evaluation alongside a clinical trial in the UK
Source: BMJ Open. 2017 Nov 26;7(11):e016211. doi: 10.1136/bmjopen-2017-016211 (PMC5719311; doi:10.1136/bmjopen-2017-016211)
Supplement: Author's manuscript [file bmjopen-2017-016211.draft_revisions.pdf]

BMJ Open is committed to open peer review. As part of this commitment we make the peer review history of every article we publish publicly available.

When an article is published we post the peer reviewers' comments and the authors' responses online. We also post the versions of the paper that were used during peer review. These are the versions that the peer review comments apply to.

The versions of the paper that follow are the versions that were submitted during the peer review process. They are not the versions of record or the final published versions. They should not be cited or distributed as the published version of this manuscript.

BMJ Open is an open access journal and the full, final, typeset and author-corrected version of record of the manuscript is available on our site with no access controls, subscription charges or pay-per-view fees (http://bmjopen.bmj.com).

If you have any questions on BMJ Open's open peer review process please email editorial.bmjopen@bmj.com

BMJ Open

# A preferred intensity exercise programme for young people with depression: a cost-effectiveness study.

| | |
|---|---|
| Journal: | *BMJ Open* |
| Manuscript ID | bmjopen-2017-016211 |
| Article Type: | Research |
| Date Submitted by the Author: | 31-Jan-2017 |
| Complete List of Authors: | Turner, David; University of East Anglia, Public Health and Primary Care<br>Carter, Timothy; University of Nottingham School of Health Sciences<br>Sach, Tracey; University of East Anglia, Public Health and Primary Care, Norwich Medical School<br>Guo, Boliang; Institute of Mental Health, Psychiatry<br>Callaghan, Patrick; University of Nottingham, School of Health Sciences |
| <b>Primary Subject Heading</b>: | Health economics |
| Secondary Subject Heading: | Mental health |
| Keywords: | HEALTH ECONOMICS, Depression & mood disorders < PSYCHIATRY, Child & adolescent psychiatry < PSYCHIATRY |
| | |

SCHOLARONE™
Manuscripts

A preferred intensity exercise programme for young people with depression: a cost-effectiveness study.

David Turner[1]*, Tim Carter[2], Tracey Sach[1], Boliang Guo[3], Patrick Callaghan[2]

[1] Norwich Medical School, University of East Anglia, UK

[2] School of Health Sciences, University of Nottingham, UK

[3] Division of Psychiatry & Applied Psychology, University of Nottingham, UK

*Corresponding author: David Turner.

Tel: 01603-593960.

Fax: 01603 593752

e-mail: David.A.Turner@uea.ac.uk

E-mails of other authors

Timothy Carter: Timothy.Carter@nottingham.ac.uk

Tracey Sach: T.Sach@uea.ac.uk

Boliang Guo: Boliang.Guo@nottingham.ac.uk

Patrick Callaghan: Patrick.Callaghan@nottingham.ac.uk

For peer review only



For peer review only - http://bmjopen.bmj.com/site/about/guidelines.xhtml

**Abstract**

**Objectives:** To assess the cost-effectiveness of preferred intensity exercise programme for young people with depression compared to a treatment as usual control group.

**Design:** A ‘within trial’ cost-effectiveness and cost-utility analysis conducted alongside a randomised controlled trial. The perspective of the analysis was NHS and social services.

**Setting:** The intervention was provided in a community leisure centre setting.

**Participants:** 86 young people aged 14 to 17 years attending Tier 2 and Tier 3 Child and Adolescent Mental Health (CAMHS) outpatient services presenting with depression.

**Interventions**: The intervention comprised 12 separate sessions of circuit training over a 6 week period. Sessions were supervised by a qualitied exercise therapist. Participants also received treatment as usual. The comparator group received treatment as usual.

**Results:** We found improvements in the Children’s Depression Inventory-2 (CDI-2) and estimated cost-effectiveness at £80 per point improvement in CDI-2 for the exercise group compared to control. We found no evidence of differences in QALYs between groups.

**Conclusions:** There is evidence that exercise can be an effective intervention for adolescents with depression and the current study shows that preferred intensity exercise could also represent a cost-effective intervention in terms of the CDI-2.

Keywords: depression; health economic evaluation; adolescence; cost-effectiveness.

For peer review only



For peer review only - http://bmjopen.bmj.com/site/about/guidelines.xhtml

Strengths and Limitations of this study

- Economic analysis conducted alongside a randomised trial of an exercise intervention for adolescents with depression.
- Trial was conducted in 86 participants and constitutes the largest trial of its type.
- This study uses costs and effectiveness data collected directly from the trial population to estimate the cost-effectiveness of an exercise intervention for adolescents with depression.
- The small sample size at follow-up limits the strength of the conclusions made.

**Funding statement**

The study was funded by the National Institute of Health Research (NIHR), Research for Patient Benefit Programme, grant reference number: PB-PG-1208-18097.

For peer review only

**Background**

Depression is highly prevalent in adolescence [1] with the numbers of reported cases doubling between the mid-1980s and 2000s. [2] Among community samples of adolescents, the prevalence of Major Depressive Disorder (MDD) is reported to be between 4%-8% [3]. Additionally, it is estimated that 12% of children and adolescents may have sub threshold symptoms of depression [4]. There is some evidence to suggest that exercise may have beneficial effects on depression in young people.[5-8] A recent feasibility RCT compared a moderate to strenuous exercise intervention to stretching for adolescents diagnosed with MDD.[9] Statistically significant reductions in depression scores were observed for both groups. However, the study was limited by a small sample size (30 participants initially with 15 completing 12-month follow-up). For this reason a randomised controlled trial was designed and conducted to determine the effectiveness of a preferred intensity exercise intervention on the depressive symptoms of adolescents with depression.[10]

With growing concern as to the affordability of healthcare, particularly in the context of the NHS budget, it is important to consider the value for money afforded by any intervention in terms of both its costs and effectiveness. Furthermore, the costs of child and adolescent mental health can be substantial with a recent study estimating total annual costs of emotional disorders in the UK at £1,165 per person in 2007/8 prices.[11] Individuals treated for depression during childhood can continue to incur substantial costs into adulthood.[12] These considerations should be contemplated when making resource allocations decisions. However, there has been limited and inconclusive evidence looking at the health economic case for interventions for child or adolescent depression. One study we are aware of looked at an exercise related intervention.[13] This was a cost-utility study of a dance intervention for adolescent girls with internalizing problems. The dance intervention was considered cost-effective (US$3,830 per QALY). There have also been studies that conducted economic evaluations of adolescents with depression using non exercise based interventions. An economic evaluation was conducted in the USA alongside a randomised controlled trial for



For peer review only - http://bmjopen.bmj.com/site/about/guidelines.xhtml

subjects with a major diagnosis of depression.[14 15] Groups received either placebo, fluoxetine, cognitive behaviour therapy (CBT), or a combination of fluoxetine and CBT. Researchers found that combination therapy with fluoxetine and CBT was highly likely to be cost-effective at 6 weeks. A study in the UK evaluated CBT in addition to serotonin reuptake inhibitors (SSRI) with normal clinical care.[14] The comparator was SSRI plus normal clinical care alone. This study found only a 30% probability that CBT and SSRI would be more cost-effective than SSRI alone. This evidence is limited and only one study was conducted in the UK. For these reasons we conducted an economic evaluation alongside the aforementioned clinical trial.[10] The aim of this economic evaluation was to examine the cost-effectiveness of a preferred exercise intervention in addition to usual care compared to usual care alone. This article describes the methods used to conduct this economic analysis. We present the results in terms of both costs and effects for the brief exercise intervention compared to standard care.

## Methods

### Randomised controlled trial

The current economic evaluation was conducted as an integral part of a randomised controlled trial.[10] In brief, this study compared a preferred intensity exercise intervention, in addition to treatment as usual (TAU), with a TAU only control group. The sample was drawn from young people attending Tier 2 and Tier 3 Child and Adolescent Mental Health (CAMHS) outpatient services in Nottingham City and Nottinghamshire County, UK. To be included in the study participants needed to be: adolescents aged between 14 and 17; in receipt of treatment from a health or social care professional for depression; and scoring 14 or above on the Children's Depression Inventory-2 (CDI-2). [15] The study comprised 86 participants, who were individually randomised to study groups by means of sequentially numbered opaque sealed envelopes. The intervention package consisted of a maximum of 12



For peer review only - http://bmjopen.bmj.com/site/about/guidelines.xhtml

sessions, delivered over a 6 week period. Sessions consisted of aerobic exercise in the form of circuit training; tailored to the exercise preferences of participants. Exercise sessions were scheduled for 60 minutes and were given in groups with a maximum size of 10 participants. Participants were followed-up for 6 months. Informed written consent was obtained from the legal guardians of those under the age of 16, alongside the young person's assent. Informed written consent was obtained directly from those 16 years of age and over. Ethical approval was received from Nottingham Research Ethics Committee on 18/07/11 (REC reference: 11/EM/)

## Costs

The perspective was that of the NHS and social services. All costs were for the year 2012/13, measured in UK pounds sterling. As the time frame of the analyses was less than one year neither costs or outcomes were discounted. The attendance of participants in the treatment group was recorded at each session. Sessions were run by two members of research staff. As this service was run from a CAMHS unit, data on staff and non-staff overheads relevant to CAMHS were taken from a published source of health care unit cost data. [16] Actual costs were also recorded for other resource items required to provide the sessions, e.g. the cost of room bookings.

Health and social care resource use data was collected using the client service receipt inventory (CSRI). This is a comprehensive inventory of resource use that has been widely used in economic evaluations of mental health interventions and can be adapted to fit individual contexts.[17 18] The CSRI was completed at baseline and at the 6-month follow-up period by means of a face-to-face interview conducted by a member of the study team who was blinded to study group. The time frame used was the last 6-months for all questions. Data were collected on the following: medicines; inpatient stays; use of other hospital services; contacts with health care practitioners; and social care. The CSRI also covered



For peer review only - http://bmjopen.bmj.com/site/about/guidelines.xhtml

resource use relating to overnight stays in children's homes and foster care alongside other services used by the respondent's family as a result of the young person's behavioural or mental health problems.

Resources identified were valued using the unit costs identified in Table 1. Medicines were costed using the British National Formulary (BNF), [19] accessed March 2014. Prices obtained were deflated to 2012/13 using the consumer price index (CPI). Where it was unclear what medicine was prescribed, a clinical expert (practising GP) was consulted about typical prescribing for the reason given by the respondent. For inpatient stays, NHS reference costs were used.[20] Where a stay in a mental health facility was recorded, a cost per day of £611 was used. Where the reason for admission was clear, costs were based on appropriate NHS reference costs. Where the reason was not clear, we used weighted average costs. A&E and outpatient costs were taken from NHS reference costs.[20] For mental health related A&E contacts, the cost used was that of the A&E Mental Health Liaison services. For other A&E visits, a weighted average of all non-admitted A&E contacts was used. Mental health outpatient contacts were estimated using a weighted average of outpatient and community CAMHS contacts. For other outpatient visits we used the cost for a paediatrics outpatient visit.

A range of contacts with community health care professionals were recorded by the CSRI. The unit costs used for these are also shown in Table 1. Where necessary, assumptions on duration of contact were made. The modified CSRI asked for contacts with a number of different types of counsellor. As no further details were available we assumed that all counselling services had the same cost, taken from a published source.[16] The cost of a visit to a clinical psychologist was based on an assumed duration of contact of 30 minutes [16]. The cost of a social worker was obtained from a published source.[16] It was not clear what the



For peer review only - http://bmjopen.bmj.com/site/about/guidelines.xhtml

duration of contacts would be so an assumption of 20 minutes was used. Individuals were asked for any overnight stays in the last six months in either a children's home, foster carer, or any other residential placement. One individual reported a stay in the 'other residential placement' category, and only in the baseline period. In the absence of any other information we assumed this was equivalent to foster care and used a cost of £91 per day [16].

**Outcome measures**

Two separate measures of outcome were used for the economic evaluations. Firstly, we used the primary outcome measure from the clinical study, the Children's Depression Inventory (CDI-2) – a 28-item self-report questionnaire that assesses the severity of current/recent depressive symptoms in young people aged 7 to 17 over the preceding two weeks.[15] Questions on the CDI-2 have 3 possible responses: 0, corresponding to no symptoms; 1, corresponding to probably or mild symptoms; and 2, corresponding to definite or marked symptoms. This gives a range of scores from 0 to 56 with higher scores representing higher depressive symptom severity. A score of 14 and above is considered to indicate clinical levels of depression.[15] The CDI-2 was used in a cost-effectiveness study to estimate cost per point change in CDI-2. We also carried out a cost-utility study estimating the cost per quality adjusted life year (QALY) generated by the exercise intervention. QALYs were estimated using the five level EuroQol (EQ-5D-5L) instrument.[21] This was scored using a published scoring algorithm .[22] Both outcome measures were administered at base-line, post-intervention (approximately 6 weeks after commencement of exercise) and at the 6-month follow-up.

**Analysis**

QALYs were estimated for the follow-up period using 'area under the curve'. To do this we assumed a linear relationship between the three data collection points (baseline, post-intervention, and follow-up). QALYs were estimated using the actual time of each of the



For peer review only - http://bmjopen.bmj.com/site/about/guidelines.xhtml

three data collection points so length of time could differ between respondents. Regression based methods were used to allow for differences in baseline characteristics. [23] Differences between the intervention and the control group for both costs and outcomes were estimated using seemingly unrelated regression (using sureg command in STATA). Costs were estimated controlling for: study group; baseline EQ-5D-5L; and baseline total cost. Estimates for differences in CDI-2 controlled for: study group; baseline CDI-2; and time from baseline to final follow-up. Estimates for differences in QALY controlled for: study group; baseline EQ-5D; and time from baseline to follow-up. These estimates were used to estimate incremental cost-effectiveness ratios. Bootstrapping with 2000 samples was used to estimate cost-effectiveness acceptability curves (CEACs).[24]

For costs and QALYs the base-case analyses are presented as complete case analyses, i.e. only including those individuals for whom we had baseline, 6-week, and 6-month follow up data from. All individuals who had follow-up resource use data had EQ-5D scores as these two measures were taken together. However, there were four individuals for whom we had almost complete data except for one missing EQ-5D score. We imputed QALY scores for these 4 data points using multiple imputation and included these individuals in the baseline analysis. In addition to this we included a sensitivity analysis where we imputed missing data where the follow-up data were not available. All analysis were conducted in SPSS version 23 and Stata 11.

## Results

There were 86 individuals recruited to the study, 42 and 44 in the control and exercise groups respectively. These individuals completed a baseline resource use questionnaire. Of these, 42 (17 controls and 25 in exercise group) completed the follow-up resource use questionnaire. Descriptive characteristics are given for all participants and for those in the



For peer review only - http://bmjopen.bmj.com/site/about/guidelines.xhtml

complete case analysis (Table 2). Baseline characteristics are similar between groups for both the full data set and the health economic complete case analysis. There were no statistically significant differences between the control and intervention group for either the full data set or the complete health economics data.

**Costs**

The total time required to provide exercise sessions, including set up and travel, was two hours. The cost, including overheads, for two individuals for two hours was £129, additionally, there was a charge of £23 for use of space in the leisure centre. This gave an estimate of £152 for the cost of a group session. There were 44 individuals randomised to the intervention group. Participants joined one of seven different exercise groups and each group had 12 scheduled sessions. This gave 82 sessions (two sessions were cancelled due to low numbers) and hence a total estimated cost for the intervention of £12,464. Of those in the exercise group, 8 did not attend any sessions. There were a total of 277 attendances by the other individuals. This gave an average cost per person per session of £45.

The NHS and social care costs incurred by participants are given in Table 3 for the complete case analysis. Estimated average costs at baseline were £3,312 and £3,280 for control and intervention groups respectively. These differences were not statistically significant. Costs were much lower in the follow-up period at £1,301 for the control group and £1,889 for the intervention group (this includes £351 for the cost of the intervention). The difference in costs between groups was £589; with a 95% confidence interval of -£507 to £1,685.

**Outcomes**

The outcome measures used in this study are shown in Table 4. EQ-5D-5L scores increase between baseline and follow-up in both groups. It can also be seen that the CDI-2 scores



For peer review only - http://bmjopen.bmj.com/site/about/guidelines.xhtml

decrease (improve) in each group. Estimated QALY was higher in the control group, however, this group has a higher starting EQ-5D-5L value, so mean unadjusted QALYs would favour this group. Therefore regression methods were used to estimate the QALY effect due to being in the intervention group. The results for this can be seen in Table 5. The estimate difference in QALY score was -0.002 (95% CI: -0.04 to 0.04, p=0.93) for the complete case analysis. This indicates that we found no difference in QALY between groups. The estimated difference between baseline and follow-up CDI-2 is -4.6, indicating that the intervention groups CDI-2 score improved by 4.6 additional points compared to the control group.

Estimates of cost effectiveness are also shown in Table 5. To be consistent with the QALY ICERs and to more meaningful we have changed the sign of the difference in CDI-2 scores when calculating the ICER. This is because a decrease in the CDI-2 represents an improvement in symptoms so is a positive benefit. Because of the small numbers for whom we have data the results are subject to considerable uncertainty and should be treated with caution. No differences were statistically significantly in the complete case analysis, however the CDI-2 scores were significantly different in the imputed analysis. The cost per point improvement in the CDI-2 was £80. As there is a very small decrease in QALYs the ICERs are negative though with considerable uncertainty. CEACs for these results are shown in Figures 1 and 2 for the complete case analysis. Figure 1 indicates the probability that the intervention is cost-effective at different values of a point improvement in the CDI-2. There is a 50% probability that the intervention is cost-effective at values of a point change in CDI-2 of approximately £80. Due to considerable uncertainty and the very small incremental effect on QALYs the CEAC for the cost/QALY analysis (Figure 2) shows much lower probability that the intervention is cost-effective at around 20 to 25% at a cost per QALY of £20,000 to £30,000. As a sensitivity analysis we also calculated cost-effectiveness ratios using imputed



For peer review only - http://bmjopen.bmj.com/site/about/guidelines.xhtml

costs. The main difference here is that the estimated cost difference is lower. This results in a cost per point change in CDI-2 of £40. Results for the QALY analysis are similar to those of the complete case analysis.

For peer review only



For peer review only - http://bmjopen.bmj.com/site/about/guidelines.xhtml

## Discussion

We found that the intervention lead to improvements in the CDI-2 score, costing £80 and £40 per point improvement in CDI-2 for the complete case and imputed data respectively. Mean costs in the follow-up period were higher for the intervention group, mainly due to the cost of the intervention. We found no evidence that the intervention was associated with improvements in QALYs. This study comprised the largest trial of its type in a clinical population of adolescents with depression. Additionally, as the clinical study had a parallel economic evaluation we were able to directly assess the costs and cost-effectiveness of this intervention. Data on resource use was collected in face-to-face interviews ensuring that that completion of individual questions was high for those who completed follow-up. Additionally, outcomes measures and resource use questions were collected in the same interview ensuring that where we had resource use data we also had EQ-5D-5L data.

One weakness of the current study was that there was considerable loss to follow-up at the final time point. This was less of an issue for the study primary outcome measure, which was based on CDI-2 at the 6-week follow-up, but was more problematic for the economic evaluation. Only those who completed the final assessment would have completed the follow-up CSRI so only these participants could be included in the complete case analysis. This meant that only 49% cases were included in the complete case analysis for the cost-effectiveness study. We used multiple imputation to estimate costs and QALYs for missing cases. This produced a lower estimate of the cost per point change in CDI-2. Another potential disadvantage was that there were often delays between baseline data collection and starting the intervention. In the control group there were similar delays. This meant that the total length of follow-up varied between participants. Mean follow-up was 36 weeks in both groups. As the period of recall was 26 weeks for the CSRI this did not cover all costs



For peer review only - http://bmjopen.bmj.com/site/about/guidelines.xhtml

between baseline and follow-up in all participants. This may have understated total costs in the follow-up period as average 6-month costs are declining over time.

The study estimated that differences between baseline and follow-up CDI-2 were greater in the intervention group. This difference was -4.6 for the complete case analysis and -4.9 for the imputed data. There is limited evidence of estimating QALY in adolescents with depression. In an economic evaluation of a dance intervention for adolescent girls with internalizing problems QALYs were estimated using the Health Utilities Index (HUI3).[13] This intervention was considered cost-effective (US$3,830 per QALY), however there were differences in how the QALY effect was calculated, i.e., this used unadjusted differences in QALY compared to baseline QALY whereas our study used regression based methods to allow for differences in baseline characteristics. A US study estimated QALYs by means of depression free days. [25 26] Depression free days were given a value of 1.0 and days with depression were given a value of 0.6. The authors estimated QALYs by combining these values with data on the number of days depression free to estimate QALYs. We are aware of one published study that evaluated QALYs in adolescents with depression using the three level EQ-5D-3L.[14] This study found no difference in QALYs between groups and did not find the intervention to be cost effective.

The fact that the main clinical paper[10] and the current economic evaluation indicated differences in CDI-2 scores but not in the EQ-5D or QALYs may therefore be due to a number of factors. Firstly, the EQ-5D may be unsuitable for use in adolescents with depression, i.e. it may be insensitive to changes that can be detected by depression specific measures. We found only one published study that evaluated the performance of the EQ-5D in adolescents with depression [27]. This study found a statistically significant correlation between EQ-5D and depression specific outcome measures, though this relationship was stated to be weak. However, there is considerable evidence looking at the use of EQ-5D in



For peer review only - http://bmjopen.bmj.com/site/about/guidelines.xhtml

adults with depression. A recent systematic review found 14 studies which evaluated the performance of the EQ-5D in patients with depression and/or anxiety [28]. The mean age of participants in these studies ranged from 39 to 49 years of age. The authors of this systematic review concluded that the EQ-5D showed good construct validity and responsiveness for people with depression. These studies provide limited evidence for the use of EQ-5D in adolescents with depression.

Another factor may be that the current study used the 5 level version of the EQ-5D (EQ5D-5L) [21]. All the currently available literature evaluating the EQ-5D in depression uses the 3-level version (EQ5D-3L) [29]. It may be that the 5-level version performs less well in people with depression. An alternative explanation for the small non-significant estimate of the QALY effect of the intervention may simply have been that the sample size was insufficient to detect differences in this generic measure, which we would expect to be less sensitive than the depression specific score. Alternatively, there may be differences in depression related symptoms but these may not be translated into improvements in overall health related quality of life. Further research using and evaluating the EQ-5D-5L in adolescents would be beneficial.

The current study suggests that the intervention can generate improvements in the CDI-2 at approximately £80 per point improvement. A lower estimate of £40 per point change in CDI-2 was also obtained when missing costs were imputed. This may represent a cost-effective intervention but it is unclear as to what a point change in the CDI-2 should be worth. It is currently not clear what constitutes a clinically significant difference in CDI-2. Knowledge of this would contextualise the differences found in this study and help evaluate whether the differences found were worth the likely extra cost. Additionally, it is unclear how persistent any benefits generated by the intervention would be. The current study only followed



For peer review only - http://bmjopen.bmj.com/site/about/guidelines.xhtml

participants up for approximately 6 months. If benefits persisted for more than 6-months they would not be captured by the current design, which therefore may overestimate the cost per point change in CDI-2. Furthermore, the delivery of the intervention in this study was influenced because it was part of a RCT. This limited numbers available for each exercise group as some of the potentially eligible participants would have been randomised into the control group. In practice the intervention could be delivered to slightly larger groups with the same resources and hence costs per person may be lower than indicated in this trial.

## Conclusion

Mental health practitioners are committed to using  the best available evidence to guide their practice. It is important to demonstrate that any new intervention is cost-effective as well as being clinically effective. There is evidence that exercise can be an effective intervention for adolescents with depression and the current study shows that preferred intensity exercise could also represent a cost-effective intervention in terms of the CDI-2.  Incorporating exercise into the repertoire of interventions could add value to the care of children and adolescents receiving treatment and support for depression.

For peer review only



For peer review only - http://bmjopen.bmj.com/site/about/guidelines.xhtml

Table 1 Unit costs used in the analysis (UK £2012/13)

| Resource use item | Cost | Source |
|---|---|---|
| Inpatient Stays | Various | NHS reference costs [20] |
| Medicines | Various | British National Formulary [19] |
| Mental health A&E | 228 | NHS reference costs [20] |
| A&E | 115 | NHS reference costs [20] |
| O/P Mental Health | 234 | NHS reference costs [20] |
| O/P Other | 187 | NHS reference costs [20] |
| School Nurse | 14 | PSSRU [16] |
| GP | 37 | PSSRU [16] |
| Paediatrician | 187 | NHS reference costs [20] |
| Physiotherapy | 12 | PSSRU [16] |
| Clinical psychology | 68 | PSSRU [16] |
| Speech therapy | 15 | PSSRU [16] |
| Hearing specialist | 65 | NHS reference costs [20] |
| Other contacts | 28 | Weighted average of reported contacts |
| Counselling/therapist | 59 | PSSRU [16] |
| home help/care worker | 19 | PSSRU [16] |
| Social worker | 51 | PSSRU [16] |
| Overnight stay | 91 | PSSRU [16] |

For peer review only



For peer review only - http://bmjopen.bmj.com/site/about/guidelines.xhtml

Table 2. Descriptive characteristics

| Baseline characteristic | Full data set | | Complete health economics data | |
|---|---|---|---|---|
| | Control | Intervention | Control | Intervention |
| Number | 42 | 44 | 17 | 25 |
| Percentage Female | 81% | 75% | 88% | 88% |
| Percentage White | 98% | 95% | 100% | 92% |
| Age (years) | 15.4 | 15.4 | 15.5 | 15.3 |
| Baseline CDI-2 | 28.2 | 29.1 | 28.7 | 29.4 |
| Baseline EQ5D | 0.82 | 0.78 | 0.82 | 0.74 |
| Time from recruitment to follow-up (weeks) | 39 | 37 | 36 | 36 |



For peer review only - http://bmjopen.bmj.com/site/about/guidelines.xhtml

Table 3. CostsCosts at baseline and infollowin up period for control and intervention groups (£'s)

| | Baseline | | Follow-up | |
|---|---|---|---|---|
| Resource use | Control (mean(SD)) | intervention (mean(SD)) | Control (mean(SD)) | intervention (mean(SD)) |
| Medicines | 8.5 (22.7) | 28.8 (121.1) | 4.3 (11) | 9.5 (21) |
| Inpatient stays | 1142 (4203) | 1046 (4982) | 0 (0) | 44.6 (162.9) |
| Health related A&E | 40.2 (89.6) | 36.5 (85.3) | 40.2 (89.6) | 73 (205.2) |
| Other A&E | 47.4 (81.9) | 27.6 (60.1) | 20.3 (45.2) | 13.8 (50.6) |
| Mental health O/P appointments | 1432 (1892) | 983 (1317) | 771 (1281) | 983 (1525) |
| Other O/P | 44 (140.7) | 29.9 (88.4) | 110 (453.5) | 89.8 (448.8) |
| **Total costs of secondary care** | **2705 (5775)** | **2122 (5570)** | **941 (1310)** | **1204 (1632)** |
| School nurse | 11.3 (21.8) | 71.7 (272) | 22.5 (67.1) | 0 (0) |
| Health visitors | 0 (0) | 0 (0) | 0 (0) | 0 (0) |
| Cost of dental treatment | 10.3 (13.4) | 13.3 (13.9) | 13.9 (18.6) | 8.2 (14.5) |
| Cost of GP visits | 126.2 (88.8) | 126 (175) | 132.8 (107.9) | 72.5 (91.6) |
| Costs of visits to paediatrician | 44 (105.1) | 37.4 (93.5) | 0 (0) | 15 (74.8) |
| Optician visits | 5.9 (9.4) | 8 (12.9) | 1.2 (4.9) | 7.2 (24.4) |
| Physiotherapist | 2.8 (11.6) | 3.8 (16.9) | 0 (0) | 0 (0) |
| Clinical psychology | 0 (0) | 3.6 (18) | 127.1 (358.7) | 0 (0) |
| Speech therapy | 0.9 (3.6) | 0 (0) | 0 (0) | 0 (0) |
| Hearing specialist | 0 (0) | 18.2 (69) | 0 (0) | 2.6 (13) |
| Other visits to health practitioners | 37.5 (59.6) | 13.9 (39.4) | 0 (0) | 0 (0) |
| **Total costs of visits to health practitioners.** | **239 (140)** | **296 (358)** | **297.5 (428.3)** | **105.5 (117.9)** |
| Family counselling | 31.2 (88.8) | 2.4 (11.8) | 0 (0) | 0 (0) |
| Individual Counselling | 226 (282) | 316 (423) | 38.2 (142.9) | 125.1 (589.6) |
| Other Counselling | 76.4 (138) | 14.2 (49) | 13.9 (57.2) | 0 (0) |
| **Total counselling costs** | **333 (375)** | **333 (414)** | **52.1 (150.3)** | **125 (590)** |



For peer review only - http://bmjopen.bmj.com/site/about/guidelines.xhtml

| | | | | |
|---|---|---|---|---|
| Home help care worker | 0 (0) | 6.8 (34.2) | 0 (0) | 15.2 (76) |
| Social worker | 6 (24.7) | 61.2 (250) | 0 (0) | 40.8 (204) |
| After school clubs | 0 (0) | 0 (0) | 0 (0) | 0 (0) |
| Other support services | 1.1 (4.6) | 0 (0) | 0 (0) | 0 (0) |
| **Total social care** | **7.1 (24.9)** | **68 (250)** | **0 (0)** | **56 (280)** |
| Nights in children's home | 0 (0) | 0 (0) | 0 (0) | 0 (0) |
| Nights in foster care | 0 (0) | 0 (0) | 0 (0) | 0 (0) |
| Nights in other residential care | 0 (0) | 364 (1820) | 0 (0) | 0 (0) |
| **Total for residential care** | **0 (0)** | **364 (1820)** | **0 (0)** | **0 (0)** |
| **Illness related service use by family** | **19.9 (64.1)** | **67.6 (137)** | **5.6 (16.2)** | **38.4 (139.8)** |
| **Costs of intervention** | - | - | **0 (0)** | **351 (144)** |
| **Total follow up costs** | **3312 (5980)** | **3280 (6236)** | **1301 (1512)** | **1889 (1853)** |

Based on sample of 42 cases (17 controls and 25 in intervention group).



For peer review only - http://bmjopen.bmj.com/site/about/guidelines.xhtml

Table 4 EQ-5D-5L scores at different time points (SD))

| Outcome measure | Control mean (95% CI) | Intervention mean (95% CI) |
|---|---|---|
| Baseline EQ-5D-5L | 0.818 (0.741 , 0.895) | 0.744 (0.683 , 0.804) |
| Post intervention EQ-5D-5L | 0.841 (0.788 , 0.893) | 0.823 (0.761 , 0.885) |
| Follow-up EQ-5D-5L | 0.881 (0.836 , 0.926) | 0.838 (0.768 , 0.908) |
| Time in study | 36.300 (31.4 , 41.3) | 36.300 (32.6 , 40) |
| QALY | 0.594 (0.507 , 0.681) | 0.567 (0.494 , 0.641) |
| Baseline CDI-2 | 28.7 (25.1 , 32.4) | 29.4 (25.2 , 33.6) |
| Follow-up CDI-2 | 24.6 (19.3 ,29.8) | 20.4 (15.9 , 24.8) |

For peer review only



For peer review only - http://bmjopen.bmj.com/site/about/guidelines.xhtml

Table 5

| Outcome | Analysis | Incremental Cost | P | CI | Incremental effect | P | CI | ICER |
|---|---|---|---|---|---|---|---|---|
| CDI-2 | Complete Case | 370 | 0.47 | (-661 , 1401) | -4.6 | 0.11 | (-10.4 , 1.1) | 80 |
| difference | Imputed | 197 | 0.45 | (-324 , 717) | -4.9 | 0.00 | (-7.7 , -2) | 40 |
| Complete | Complete Case | 370 | 0.47 | (-661 , 1401) | -0.002 | 0.93 | (-0.04 , 0.04) | Negative |
| case | Imputed | 197 | 0.45 | (-324 , 717) | -0.001 | 0.94 | (-0.02 , 0.02) | Negative |



For peer review only - http://bmjopen.bmj.com/site/about/guidelines.xhtml

Figure 1. Cost-effectiveness Acceptability Curve (CEAC) for cost per point change in CDI-2

For peer review only



For peer review only - http://bmjopen.bmj.com/site/about/guidelines.xhtml

Figure 2. Cost-effectiveness Acceptability Curve (CEAC) for cost per QALY analysis

For peer review only



For peer review only - http://bmjopen.bmj.com/site/about/guidelines.xhtml

The authors declare that they have no competing interests

Authors contributions: PC and TC lead the clinical trial. BG was the trial statistician. TS designed the original economic analysis. DT amended data collection forms and conducted the economic analysis. DT drafted the manuscript. All authors commented on the manuscript and inputted into relevant sections. All authors read and approved the final manuscript. DT takes responsibility for integrity of the health economics analysis reported here.

Acknowledgements

The study was funded by the National Institute of Health Research (NIHR), Research for Patient Benefit Programme, grant reference number: PB-PG-1208-18097. The funders played no role in the collection, analysis, and interpretation of data or in the writing of this manuscript.

The authors acknowledge the contribution of all study participants, and their parents, staff of the Child and Adolescent Mental Health Services at Nottinghamshire Healthcare NHS Trust; without whom this study would not have been possible.

For peer review only

## References


1. Thapar A, Collishaw S, Pine DS, et al. Depression in Adolescence. Lancet 2012;**379**(9820):1056-67.
2. Collishaw S, Maughan B, Natarajan L, et al. Trends in adolescent emotional problems in England: a comparison of two national cohorts twenty years apart. Journal of Child Psychology and Psychiatry 2010;**51**(8):885-94.
3. Birmaher B, Brent D, Bernet W, et al. Practice parameter for the assessment and treatment of children and adolescents with depressive disorders. 1503-26 2007;**46**(11):1503-26.
4. Wesselhoeft R, Sorensen MJ, Heiervang ER, et al. Subthreshold depression in children and adolescents - a systematic review. . Journal of Affective Disorders 2013;**151**(1):7-22.
5. Babiss LA, Gangwisch JE. Sports participation as a protective factor against depression and suicidal ideation in adolescents as mediated by self-esteem and social support. . journal of Development and Behavioural Pediatrics 2009;**30**(5):376-84.
6. Jerstad SJ, Boutelle KN, Ness KK, et al. Prospective reciprocal relations between physical activity and depression in female adolescents. . Journal Of Consulting And Clinical Psychology 2010;**78**(2):268-72.
7. Neissaar I, Raudsepp L. Changes in Physical Activity, Self-Efficacy and Depressive Symptoms in Adolescent Girls. . Pediatric Exercise Science 2011;**23**(3):331-43.
8. Sund AM, Larsson B, Wichstrøm. Role of physical and sedentary activities in the development of depressive symptoms in early adolescence. . Social Psychiatry And Psychiatric Epidemiology 2011;**46**(5):431-41.
9. Hughes CW, Barnes S, Barnes C, et al. Depressed Adolescents Treated with Exercise (DATE): A pilot randomized controlled trial to test feasibility and establish preliminary effect sizes. . Mental Health and Physical Activity 2013;**6**(2):119-31.
10. Carter T, Guo B, Turner D, et al. Preferred intensity exercise for adolescents receiving treatment for depression: a pragmatic randomised controlled trial BMC Psychiatry 2015;**15**(1):247.
11. Snell T, Knapp M, Healey A, et al. Economic impact of childhood psychiatric disorder on public sector servicess in Britain: estimates from national survey data. Journal of Child Psychology and Psychiatry 2013;**54**(9):977-85.
12. Knapp M, McCrone P, Fombonne E, et al. The Maudsley long-term follow-up of child and adolescent depression: 3. Impact of comorbid conduct disorder on service use and costs in adulthood. British journal of Psychiatry 2002;**180**:19-23.
13. Philipsson A, Duberg A, Mőller M, et al. Cost-utility analysis of a dance intervention for adolescent girls with internalising problems Cost Effectiveness and Resource Allocation 2013;**11**(4).
14. Byford S, Barrett B, Roberts C, et al. Cost-effectiveness of selective serotonin reuptake inhibitors and routine specialist care with and without cognitive–behavioural therapy in adolescents with major depression. British journal of Psychiatry 2007 **191**:521-27.
15. Kovacs M, Staff. M. *Children's Depression Inventory 2nd Edition (CDI 2) Technical manual.*: Multi-Health Systems, 2011.
16. Curtis L, editor. *Unit Costs of Health and Social Care 2013*. Canterbury: University of Kent, PSSRU, 2013.
17. Beecham J, Knappp M. Costing psychiatric interventions. In: Thornicroft G, ed. Measuring mental health needs. 2nd ed. London: Gaskell, 2001.
18. Sleed M, Eccleston C, Beecham J, et al. The economic impact of chronic pain in adolescence: methodological considerations and a preliminary costs-of-illness study. Pain 2005;**119**(1-3):183-90.
19. Joint Formulary Committee. British National Formulary (online). Secondary Joint Formulary Committee. British National Formulary (online) 2014. http://www.medicinescomplete.com.
20. NHS Reference costs. In: Health Do, ed. London: Department of Health, 2013.
21. Herdman M, Gudex C, Lloyd A, et al. Development and preliminary testing of the new five-leve version of EQ-5D (EQ-5D-5L). Quality of Life Research 2011;**20**:1727-36.
22. Devlin N, Shah K, Feng Y, et al. Valuing Health-Related Quality of Life: An EQ-5D-5L Value Set for England. 2016; 2016(14/10/2016). https://www.ohe.org/publications/valuing-health-related-quality-life-eq-5d-5l-value-set-england.
23. Manca A, Hawkins N, Sculpher M. Estimating mean QALYs in trial based cost-effectivenss analysis: the importance of controlling for baseline utility. Health Economics 2005;**14**(5):487-96.
24. Löthgren M, Zethraeus N. Definition, interpretation and calculation of cost-effectiveness acceptability curves. . Health Economics 2000;**9**(7):623-30.
25. Domino ME, Burns BJ, silva SG, et al. Cost-Effectiveness of Treatments for Adolescent Depression: Results From TADS. American Journal of Psychiatry 2008;**165**(5):588-96.
26. Domino ME, Foster EM, Vitiello B, et al. Relative cost-effectiveness of treatments for adolescent derpession: 36-week results from the TADS randomized trial Journal of the American Academy of Child and Adolescent Psychiatry 2009;**48**(7):711-20.




For peer review only - http://bmjopen.bmj.com/site/about/guidelines.xhtml

27. Byford S. The validity and responsiveness of the EQ-5D measure of health-related quality of life in aan adolescent population with persistent major depression. Journal of Mental Health 2013;**22**(2):101-10.
28. Brazier J, Connell J, Papaionnou D, et al. A systematic review, psychometric analysis and qualitative assessment of generic preference-based measures of health in mental health populations and the estimation of mapping functions from widely used specific measures. Health Technology Assessment 2014;**18**(34).
29. Brooks R. EuroQol: the current state of play. Health Policy 1996;**37**(1):53-72.

For peer review only



For peer review only - http://bmjopen.bmj.com/site/about/guidelines.xhtml

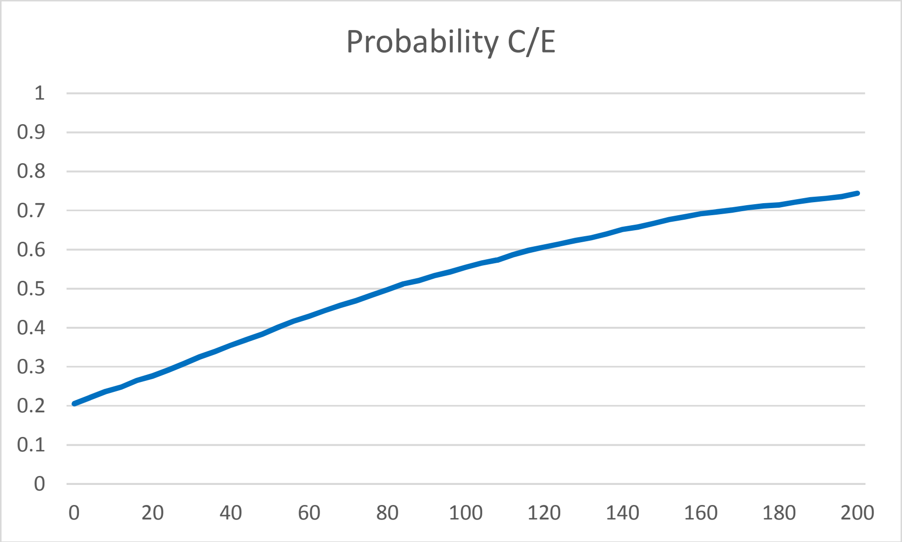


eer review only

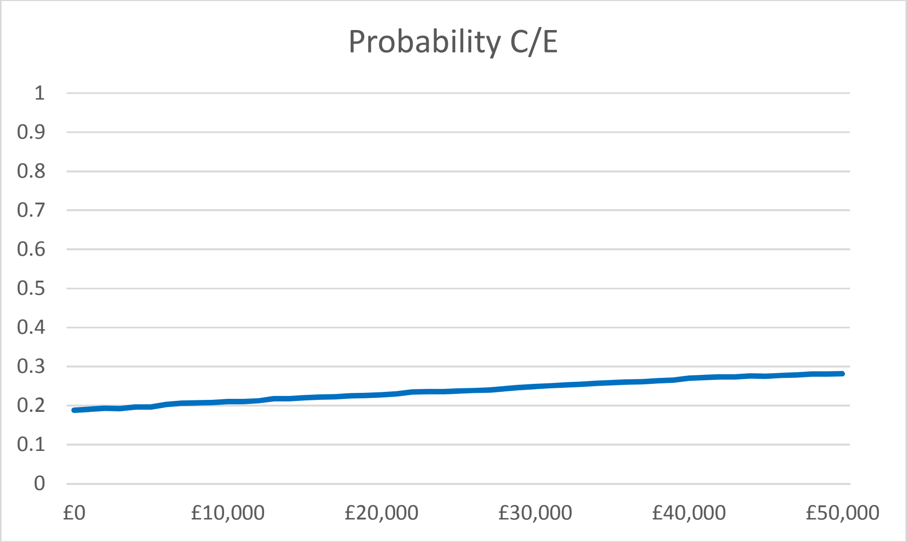


For peer review only

CHEERS Statement

| Title and Abstract | Item | Recommendation | Reported on page number/line number |
|---|---|---|---|
| Title | 1 | Identify the study as an economic evaluation or use more specific terms such as 'cost-effectiveness analysis', and describe the interventions compared. | Page 1<br>Specifies cost-effectiveness and names intervention |
| Abstract | 2 | Provide a structured summary of objectives, perspective, setting, methods (including study design and inputs), results (including base case and uncertainty analysis), and conclusions. | Page 2, structured abstract is provided. |
| Introduction | | | |
| Background and objectives | 3 | Provide an explicit statement of the broader context for the study | Yes, page 4-5 |
| | | Present the study question and its relevance for health policy or practice decisions. | Yes, page 4-5 |
| Methods | | | |
| Target population and subgroups | 4 | Describe the characteristics of the base case population and subgroups analysed, including why they were chosen. | Yes, page 5<br>Currently gives details of study population<br>There is no subgroup analysis |
| Setting and location | 5 | State relevant aspects of the system(s) in which the decisions(s) need(s) to be made. | Yes, page 5<br>Tier 2 and Tier 3 CAMHS outpatient services |
| Study perspective | 6 | Describe the perspective of the study and relate this to the costs being evaluated. | Yes, page 6 |
| Comparators | 7 | Describe the interventions or strategies being compared and state why they were chosen. | Page 5-6 |
| Time Horizon | 8 | State the time horizon(s) over which costs and consequences are being evaluated and say why appropriate. | Page 6 |
| Discount rate | 9 | Report the choice of discount rate(s) used for costs and outcomes and say why appropriate. | Page 6 |
| Choice of health outcomes | 10 | Describe what outcomes were used as the measure(s) of benefit in the evaluation and their | Page 8<br>Cost per point change in CDI-2 and cost/QALY |

For peer review only

| | | | |
|---|---|---|---|
| | | relevance for the type of analysis performed | |
| Measurement of effectiveness | 11a | *Single study-based estimates:* Describe fully the design features of the single effectiveness study and why the single study was a sufficient source of clinical effectiveness data. | Page 5-6 – randomised controlled trial section of methods |
| | 11b | *Synthesis-based estimates:* Describe fully the methods used for identification of included studies and synthesis of clinical effectiveness data. | NA |
| Measurement and valuation of preference based outcomes | 12 | If applicable, describe the population and methods used to elicit preferences for outcomes. | NA |
| Estimating resources and costs | 13a | *Single study-based economic evaluation:* Describe approaches used to estimate resource use associated with the alternative interventions. Describe primary or secondary research methods for valuing each resource item in terms of its unit cost. Describe any adjustments made to approximate to opportunity costs. | Cost section Page 6 to 8 |
| | 13b | *Model-based economic evaluation:* Describe approaches and data sources used to estimate resource use associated with model health states. Describe primary or secondary research methods for valuing each resource item in terms of its unit cost. Describe any adjustments made to approximate to opportunity costs. | NA |
| Currency, price date, and conversion | 14 | Report the dates of the estimated resource quantities and unit costs. Describe methods for adjusting estimated unit costs to the year of reported costs if necessary. Describe methods for converting costs. | Page 6 |
| Choice of model | 15 | Describe and give reasons for the specific type of decision-analytical model used. Providing a figure to show model structure is strongly recommended. | NA |
| Assumptions | 16 | Describe all structural or other assumptions underpinning the decision-analytical model. | NA |

For peer review only

| | | | |
|---|---|---|---|
| Analytical methods | 17 | Describe all analytical methods supporting the evaluation. This could include methods for dealing with skewed, missing, or censored data; extrapolation methods; methods for pooling data; approaches to validate or make adjustments (such as half cycle corrections) to a model; and methods for handling population heterogeneity and uncertainty. | Page 8 and 9 – Analysis section |
| Results | | | |
| Study parameters | 18 | Report the values, ranges, references, and, if used, probability distributions for all parameters. Report reasons or sources for distributions used to represent uncertainty where appropriate. Providing a table to show the input values is strongly recommended. | NA |
| Incremental costs and outcomes | 19 | For each intervention, report mean values for the main categories of estimated costs and outcomes of interest, as well as mean differences between the comparator groups. If applicable, report incremental cost-effectiveness ratios. | Table 3 and cost section, page 10. |
| Characterising uncertainty | 20a | *Single study-based economic evaluation:* Describe the effects of sampling uncertainty for the estimated incremental cost and incremental effectiveness parameters, together with the impact of methodological assumptions (such as discount rate, study perspective). | Page 9-12 – results section<br>Table 4<br>Table 5<br>Figure 1<br>Figure 2 |
| | 20b | *Model-based economic evaluation:* Describe the effects on the results of uncertainty for all input parameters, and uncertainty related to the structure of the model and assumptions. | NA |
| Characterising heterogeneity | 21 | If applicable, report differences in costs, outcomes, or cost-effectiveness that can be explained by variations between subgroups of patients with different baseline characteristics or other observed variability in effects that are not reducible by more | NA |

For peer review only

| | | | |
|---|---|---|---|
| | | information. | |
| Discussion | | | |
| Study findings, limitations, generalisability, and current knowledge | 22 | Summarise key study findings and describe how they support the conclusions reached.<br>Discuss limitations and the generalisability of the findings and how the findings fit with current knowledge. | Yes – Page 13<br>Yes – Page 13 to 14 |
| **Other** | | | |
| Source of funding | 23 | Describe how the study was funded and the role of the funder in the identification, design, conduct, and reporting of the analysis. Describe other non-monetary sources of support. | Yes – Page 3<br>Role of funders – page 25 |
| Conflicts of interest | 24 | Describe any potential for conflict of interest of study contributors in accordance with journal policy. In the absence of a journal policy, we recommend authors comply with International Committee of Medical Journal Editors recommendations. | Page 25 |

For peer review only

BMJ Open

# Cost-effectiveness of a preferred intensity exercise programme for young people with depression compared to standard care. An economic evaluation alongside a clinical trial in the UK

| | |
|---|---|
| Journal: | *BMJ Open* |
| Manuscript ID | bmjopen-2017-016211.R1 |
| Article Type: | Research |
| Date Submitted by the Author: | 01-Aug-2017 |
| Complete List of Authors: | Turner, David; University of East Anglia, Public Health and Primary Care<br>Carter, Timothy; University of Nottingham School of Health Sciences<br>Sach, Tracey; University of East Anglia, Public Health and Primary Care, Norwich Medical School<br>Guo, Boliang; Institute of Mental Health, Psychiatry<br>Callaghan, Patrick; University of Nottingham, School of Health Sciences |
| <b>Primary Subject Heading</b>: | Health economics |
| Secondary Subject Heading: | Mental health |
| Keywords: | HEALTH ECONOMICS, Depression & mood disorders < PSYCHIATRY, Child & adolescent psychiatry < PSYCHIATRY |
| | |

SCHOLARONE™
Manuscripts

For peer review only

# Cost-effectiveness of a preferred intensity exercise programme for young people with depression compared to standard care. An economic evaluation alongside a clinical trial in the UK

David Turner[1]*, Tim Carter[2], Tracey Sach[1], Boliang Guo[3], Patrick Callaghan[2]

[1] Norwich Medical School, University of East Anglia, UK

[2] School of Health Sciences, University of Nottingham, UK

[3] Division of Psychiatry & Applied Psychology, University of Nottingham, UK

*Corresponding author: David Turner.

Tel: 01603-593960.

Fax: 01603 593752

e-mail: David.A.Turner@uea.ac.uk

E-mails of other authors

Timothy Carter: Timothy.Carter@nottingham.ac.uk

Tracey Sach: T.Sach@uea.ac.uk

Boliang Guo: Boliang.Guo@nottingham.ac.uk

Patrick Callaghan: Patrick.Callaghan@nottingham.ac.uk



For peer review only

For peer review only - http://bmjopen.bmj.com/site/about/guidelines.xhtml

**Abstract**

**Objectives:** To assess the cost-effectiveness of preferred intensity exercise programme for young people with depression compared to a treatment as usual control group.

**Design:** A 'within trial' cost-effectiveness and cost-utility analysis conducted alongside a randomised controlled trial. The perspective of the analysis was the UK National Health Service (NHS) and social services

**Setting:** The intervention was provided in a community leisure centre setting.

**Participants:** 86 young people aged 14 to 17 years attending Tier 2 and Tier 3 Child and Adolescent Mental Health (CAMHS) outpatient services presenting with depression.

**Interventions**: The intervention comprised 12 separate sessions of circuit training over a 6 week period. Sessions were supervised by a qualified exercise therapist. Participants also received treatment as usual. The comparator group received treatment as usual.

**Results:** We found improvements in the Children's Depression Inventory-2 (CDI-2) and estimated cost-effectiveness at £61 per point improvement in CDI-2 for the exercise group compared to control. We found no evidence  that the exercise intervention led to differences in quality adjusted life years (QALYs), QALYs were estimated using the EQ-5D-5L.

**Conclusions:** There is evidence that exercise can be an effective intervention for adolescents with depression and the current study shows that preferred intensity exercise could also represent a cost-effective intervention in terms of the CDI-2.

Keywords: depression; health economic evaluation; adolescence; cost-effectiveness.

For peer review only

Strengths and Limitations of this study

- Economic analysis conducted alongside a randomised trial of an exercise intervention for adolescents with depression.
- This study uses costs and effectiveness data collected directly from the trial population to estimate the cost-effectiveness of an exercise intervention for adolescents with depression.
- Appropriate statistical methods were adopted to control for baseline characteristics and missing data
- The small sample size at follow-up limits the strength of the conclusions made

**Funding statement**

The study was funded by the National Institute of Mental Health, Research for Patient Benefit Programme, grant reference number: PB-PG-1208-18097.

**Data sharing statement**

No additional data is available. If additional clarification is required please contact corresponding author.



For peer review only - http://bmjopen.bmj.com/site/about/guidelines.xhtml

## Background

Depression is highly prevalent in adolescence [1] with the numbers of reported cases doubling between the mid-1980s and 2000s. [2] Among community samples of adolescents, the prevalence of Major Depressive Disorder (MDD) is reported to be between 4%-8% [3]. Additionally, it is estimated that 12% of children and adolescents may have sub threshold symptoms of depression.[4] There is some evidence to suggest that exercise may have beneficial effects on depression in young people.[5-8] A recent feasibility randomised controlled trial compared a moderate to strenuous exercise intervention to stretching for adolescents diagnosed with MDD.[9] Statistically significant reductions in depression scores were observed for both groups. However, the study was limited by a small sample size (30 participants initially with 15 completing 12-month follow-up). For this reason a randomised controlled trial was designed and conducted to determine the effectiveness of a preferred intensity exercise intervention on the depressive symptoms of adolescents with depression.[10 11]

With growing concern as to the affordability of healthcare, particularly in the context of the NHS budget, it is important to consider the value for money afforded by any intervention in terms of both its costs and effectiveness. Furthermore, the costs of child and adolescent mental health can be substantial with a recent study estimating total annual costs of emotional disorders in the UK at £1,165 per person in 2007/8 prices.[12] Individuals treated for depression during childhood can continue to incur substantial costs into adulthood.[13] These considerations should be contemplated when making resource allocations decisions. However, there has been limited and inconclusive evidence looking at the health economic case for interventions for child or adolescent depression. One study we are aware of looked at an exercise related intervention.[14] This was a cost-utility study of a dance intervention for adolescent girls with internalizing problems. The dance intervention was considered cost-effective as it cost US$3,830 per quality adjusted life year (QALY). There have also been studies that conducted economic evaluations of adolescents with depression using non exercise based interventions. An economic evaluation was conducted in the USA alongside

For peer review only

a randomised controlled trial for subjects with a major diagnosis of depression.[15 16] Groups received either placebo, fluoxetine, cognitive behaviour therapy (CBT), or a combination of fluoxetine and CBT. Researchers found that combination therapy with fluoxetine and CBT was highly likely to be cost-effective at 36 weeks. A study of the cost-effectiveness of a collaborative care intervention was conducted on adolescents in the USA.[17] This found the intervention to be cost-effective, at $18,239 per QALY gained. There have also been three UK studies looking at CBT in adolescents with depression. Byford and colleagues evaluated CBT in addition to serotonin reuptake inhibitors (SSRI) with normal clinical care.[18] The comparator was SSRI plus normal clinical care alone. This study found only a 30% probability that CBT and SSRI would be more cost-effective than SSRI alone. Anderson and colleagues evaluated classroom based CBT compared to usual school provision of Personal Social and Health Education. [19 20] They found no evidence that the intervention was cost-effective. Computerised CBT was also compared to a website control in a feasibility study. [21] The authors concluded a future large scale study was feasible but the study was not powered to show differences in effects.

In order to add to this literature and to aid decision making we conducted an economic evaluation alongside the aforementioned clinical trial.[11] The aim of this economic evaluation was to examine the cost-effectiveness of a preferred exercise intervention in addition to usual care compared to usual care alone. This article describes the methods used to conduct this economic analysis. We present the results in terms of both costs and effects for the brief exercise intervention compared to standard care.

## Methods

### Randomised controlled trial



For peer review only - http://bmjopen.bmj.com/site/about/guidelines.xhtml

The current economic evaluation was conducted as an integral part of a randomised controlled trial.[11] In brief, this study compared a preferred intensity exercise intervention, in addition to treatment as usual, compared to a treatment as usual only control group. The sample was drawn from young people attending Child and Adolescent Mental Health (CAMHS) outpatient services in Nottingham City and Nottinghamshire County, UK. Participants would be attending either tier 2 (typically CAMHS specialists working in primary and community care) and tier 3 (typically multidisciplinary teams in a community mental health clinic providing a more specialised service). To be included in the study participants needed to be: adolescents aged between 14 and 17; in receipt of treatment from a health or social care professional for depression; and scoring 14 or above on the Children's Depression Inventory-2 (CDI-2). [22] The study comprised 86 participants, who were individually randomised to study groups by means of sequentially numbered opaque sealed envelopes. The intervention package consisted of a maximum of 12 sessions, delivered over a 6 week period. Sessions consisted of aerobic exercise in the form of circuit training; tailored to the exercise preferences of participants. Exercise sessions were scheduled for 60 minutes and were given in groups with a maximum size of 10 participants. The sessions were preferred intensity as participants could choose the order in which they undertook different exercises, the intensity of their exercise, and when to take breaks. Participants were followed-up for 6 months. Informed written consent was obtained from the legal guardians of those under the age of 16, alongside the young person's assent. Informed written consent was obtained directly from those 16 years of age and over. Ethical approval was received from Nottingham Research Ethics Committee on 18/07/11 (REC reference: 11/EM/)

**Costs**

The perspective was that of the NHS and social services. All costs were for the year 2012/13, measured in UK pounds sterling. As the time frame of the analyses was less than one year neither costs or outcomes were discounted. The attendance of participants in the treatment group was recorded at each session. Sessions were run by two members of

For peer review only

research staff. As this service was run from a CAMHS unit, data on staff and non-staff overheads relevant to CAMHS were taken from a published source of health care unit cost data. [23] Actual costs were also recorded for other resource items required to provide the sessions, e.g. the cost of room bookings.

Health and social care resource use data was collected using the client service receipt inventory (CSRI).[24] This is a comprehensive inventory of resource use that has been widely used in economic evaluations of mental health interventions and can be adapted to fit individual contexts. Its use allows resource use patterns to be described and these can then be costed using appropriate unit costs. This instrument is designed to be adaptable and has been used in a wide number of different diseases, settings and client groups. The CSRI was completed at baseline and at the 6-month follow-up period by means of a face-to-face interview conducted by a member of the study team who was blinded to study group. The time frame used was the last 6-months for all questions. Data were collected on the following: medicines; inpatient stays; use of other hospital services; contacts with health care practitioners; and social care. The CSRI also covered resource use relating to overnight stays in children's homes and foster care alongside other services used by the respondent's family as a result of the young person's behavioural or mental health problems.

Resources identified were valued using the unit costs identified in Table 1. Medicines were costed using the British National Formulary, [25] accessed March 2014. Prices obtained were deflated to 2012/13 using the consumer price index. Where it was unclear what medicine was prescribed, a practising general practitioner (GP) was consulted about typical prescribing for the reason given by the respondent. For inpatient stays, NHS reference costs were used.[26] Where a stay in a mental health facility was recorded, a cost per day of £611 was used. Where the reason for admission was clear, costs were based on appropriate



For peer review only - http://bmjopen.bmj.com/site/about/guidelines.xhtml

NHS reference costs. Where the reason was not clear, we used weighted average costs. Accident and Emergency (A&E) and outpatient costs were taken from NHS reference costs.[26] For mental health related A&E contacts, the cost used was that of the A&E Mental Health Liaison services. For other A&E visits, a weighted average of all non-admitted A&E contacts was used. Mental health outpatient contacts were estimated using a weighted average of outpatient and community CAMHS contacts.  For other outpatient visits we used the cost for a paediatrics  outpatient visit.

A range of contacts with community health care professionals were recorded by the CSRI. The unit costs used for these are also shown in Table 1. Where necessary, assumptions on duration of contact were made. The modified CSRI asked for contacts with a number of different types of counsellor. As no further details were available we assumed that all counselling services had the same cost, taken from a published source.[23] The cost of a visit to a clinical psychologist was based on an assumed duration of contact of 30 minutes [23]. The cost of a social worker was obtained from a published source.[23] It was not clear what the duration of contacts would be so an assumption of 20 minutes was used. Individuals were asked for any overnight stays in the last six months in either a children's home, foster carer, or any other residential placement. One individual reported a stay in the 'other residential placement' category, and only in the baseline period. In the absence of any other information we assumed this was equivalent to foster care and used a cost of £91 per day [23].

**Outcome measures**

Two separate measures of outcome were used for the economic evaluations. Firstly, we used the primary outcome measure from the clinical study, the Children's Depression Inventory (CDI-2) – a  28-item self-report questionnaire that assesses  the severity of current/recent depressive symptoms in young people aged 7 to 17 over  the preceding two weeks.[22]  Questions on the CDI-2 have 3 possible responses: 0, corresponding to no symptoms; 1, corresponding to probably or mild symptoms; and 2, corresponding to definite

For peer review only

or marked symptoms. This gives a range of scores from 0 to 56 with higher scores representing higher depressive symptom severity. A score of 14 and above is considered to indicate clinical levels of depression.[22] The CDI-2 was used in a cost-effectiveness study to estimate cost per point change in CDI-2. We also carried out a cost-utility study estimating the cost per QALY generated by the exercise intervention. QALYs were estimated using the five level EuroQol (EQ-5D-5L) instrument.[27] This was scored using a published scoring algorithm .[28] Both outcome measures were administered at base-line, post-intervention (approximately 6 weeks after commencement of exercise) and at the 6-month follow-up.

**Analysis**

QALYs were estimated for the follow-up period using 'area under the curve'. To do this we assumed a linear relationship between the three data collection points (baseline, post-intervention, and follow-up). QALYs were estimated using the actual time of each of the three data collection points so length of time could differ between respondents. All individuals who had follow-up resource use data had EQ-5D scores as these two measures were taken together. However, there were four individuals for whom we had almost complete data except a single missing EQ-5D score in each case. The baseline EQ-5D score was imputed using mean value imputation for one individual. [29] Three individuals were missing EQ-5D at the 6-week follow-up and values were imputed using multiple imputation.[29] These 4 individuals were included in the base case analysis. For individuals where the follow-up questionnaire was unavailable; costs, QALYs, and difference in CDI-2 were imputed using baseline EQ-5D and CDI-2, time from randomisation to follow-up, and baseline total costs. Multiple imputation was carried out in SPSS version 23, using 50 datasets (a 'rule of thumb' is the number of datasets should equal the percentage of missing data). [30]



For peer review only - http://bmjopen.bmj.com/site/about/guidelines.xhtml

For peer review only

Regression based methods were used to allow for differences in baseline characteristics. [31] Differences between the intervention and the control group for both costs and outcomes were estimated using seemingly unrelated regression (using sureg command in STATA, version 11). Costs were estimated controlling for: study group; baseline EQ-5D-5L; and baseline total cost. Estimates for differences in CDI-2 were controlled for: study group; baseline CDI-2; and time from baseline to final follow-up. Estimates for differences in QALY were controlled for: study group; baseline EQ-5D; and time from baseline to follow-up. Estimates from the 50 imputed samples were combined using 'Rubins Rules'. [32] To estimate uncertainty associated with estimates we used bootstrap resampling with 250 replications drawn from each of the 50 imputed datasets, giving 12500 replications in total. These were used to estimate cost-effectiveness acceptability curves (CEACs).[33]

## Results

There were 86 individuals recruited to the study, 42 and 44 in the control and exercise groups respectively. These individuals completed CSRI by interview at baseline. Of these, 42 (17 controls and 25 in exercise group) completed the follow-up CSRI at interview. Descriptive characteristics are given for all participants and for those in the complete case analysis (Table 2). Baseline characteristics are similar between groups for both the full data set and the health economic complete case analysis. There were no statistically significant differences between the control and intervention group for either the full data set or the complete health economics data.

## Costs

The total time required to provide exercise sessions, including set up and travel, was two hours. The cost, including overheads, for two individuals for two hours was £129, additionally, there was a charge of £23 for use of space in the leisure centre. This gave an estimate of £152 for the cost of a group session. There were 44 individuals randomised to

For peer review only

the intervention group. Participants joined one of seven different exercise groups and each group had 12 scheduled sessions. This gave 82 sessions (two sessions were cancelled due to low numbers) and hence a total estimated cost for the intervention of £12,464. Of those in the exercise group, 8 did not attend any sessions. There were a total of 277 attendances by the other individuals. This gave an average cost per person per session of £45.

The NHS and social care costs incurred by participants are given in Table 3 for the complete case analysis. Estimated average costs at baseline were £3,312 and £3,280 for control and intervention groups respectively. These differences were not statistically significant (independent sample t-tests). Costs were much lower in the follow-up period at £1,301 for the control group and £1,889 for the intervention group (this includes £351 for the cost of the intervention). The difference in costs between groups was £589; with a 95% confidence interval of -£507 to £1,685.

**Outcomes**

The outcome measures used in this study are shown in Table 4. EQ-5D-5L scores increased between baseline and follow-up in both groups. It can also be seen that the CDI-2 scores decrease (improve) in each group. Estimated QALY was higher in the control group, however, this group has a higher starting EQ-5D-5L value, so mean unadjusted QALYs would favour this group. Therefore regression methods were used to estimate both QALY and CDI-2 differences between the exercise and control group. The results for this can be seen in Table 5. The estimated difference in QALY score was -0.0027 for the complete case analysis and 0.0019 for the imputed analysis, both with wide confidence intervals. Effects in terms of CDI-2 were slightly larger for the imputed analysis, -4.8 compared to -4.6 for the CCA. These indicate that improvements in CDI-2 score were greater in the exercise group compared to the control group.



For peer review only - http://bmjopen.bmj.com/site/about/guidelines.xhtml

Estimates of cost effectiveness are also shown in Table 5, in terms of incremental cost-effectiveness ratios (ICER). These show the additional cost of generating each additional unit of effect. To be consistent with the cost per QALY analysis we have changed the sign of the difference in CDI-2 scores when calculating the ICER. This is because a decrease in the CDI-2 represents an improvement in symptoms so is a positive benefit. Because of the small numbers for whom we have data the results are subject to considerable uncertainty and should be treated with caution. The cost per point improvement in the CDI-2 was £73. This decreased to £61 for the imputed analysis. The CCA showed a small decrease in QALYs, this meant the ICERs were negative, though with considerable uncertainty. The imputed analysis showed very small positive QALY gains and an ICER of £152,822, again with considerable uncertainty. CEACs for these results are shown in Figures 1 and 2 for the imputed analysis. Figure 1 indicates the probability that the intervention is cost-effective at different values of a point improvement in the CDI-2. There is a 50% probability that the intervention is cost-effective at values of a point change in CDI-2 of approximately £65. Due to considerable uncertainty and the very small incremental effect on QALYs the CEAC for the cost/QALY analysis shows much lower probability that the intervention is cost-effective at around 33 to 37% at a cost per QALY of £20,000 to £30,000 (Figure 2).

For peer review only

## Discussion

We found that the intervention lead to improvements in the CDI-2 score, costing £73 and £61 per point improvement in CDI-2 for the complete case and imputed data respectively. Mean costs in the follow-up period were higher for the intervention group, mainly due to the cost of the intervention. We found no evidence that the intervention was associated with improvements in QALYs. This study comprised the largest trial of its type in a clinical population of adolescents with depression. Additionally, as the clinical study had a parallel economic evaluation we were able to directly assess the costs and cost-effectiveness of this intervention. Data on resource use was collected in face-to-face interviews ensuring that that completion of individual questions was high for those who completed follow-up. Additionally, outcomes measures and resource use questions were collected in the same interview ensuring that where we had resource use data we also had EQ-5D-5L data.

One weakness of the current study was that there was considerable loss to follow-up at the final time point. This was less of an issue for the study primary outcome measure, which was based on CDI-2 at the 6-week follow-up, but was more problematic for the economic evaluation. Only those who completed the final assessment would have completed the follow-up CSRI so only 49% of participants could be included in the complete case analysis. We used multiple imputation to estimate costs and QALYs for missing cases. This produced a lower estimate of the cost per point change in CDI-2. Another potential disadvantage was that there were often delays between baseline data collection and starting the intervention. In the control group there were similar delays. This meant that the total length of follow-up varied between participants. Mean follow-up was 36 weeks in both groups. As the period of recall was 26 weeks for the CSRI this did not cover all costs between baseline and follow-up



For peer review only - http://bmjopen.bmj.com/site/about/guidelines.xhtml

in all participants. This may have understated total costs in the follow-up period as average 6-month costs are declining over time.

The study estimated that differences between baseline and follow-up CDI-2 were greater in the intervention group. We found no evidence to suggest that there were any differences in QALYs generated between groups. A number of different methods have been used to estimate QALYs. A USA study used the Health Utilities Index (HUI3) in an economic evaluation of a dance intervention for adolescent girls with internalizing problems.[14] This intervention was considered cost-effective (US$3,830 per QALY) however there were differences in how the QALY effect was calculated, i.e., this used QALYs differences from baseline utility values whereas our study used regression based methods to allow for differences in baseline characteristics. Two other US studies used depression specific outcome measures to define individuals level of depression in order to combine this with estimates of QALY effects of depression level. A US study estimated QALYs by means of depression free days.[15 16] QALYs were estimated by assigning a value of 1.0 for depression free days, and a value of 0.6 for days with depression. Wright *et al* used a similar approach where levels of depression were defined, again these were valued by utility weights for these states. [17] Both these approaches suggested differences in utility from the interventions. We are aware of four published studies, reporting on three different trials, that evaluated QALYs in adolescents with depression using the three level EQ-5D-3L.[18-21] None of these studies found that the intervention led to improvements in QALYs and did not find the intervention to be cost effective.

The fact that the main clinical paper[11] and the current economic evaluation indicated differences in CDI-2 scores but not in the EQ-5D-5L or QALYs may therefore be due to a number of factors. Firstly, the EQ-5D may be unsuitable for use in adolescents with depression, i.e. it may be insensitive to changes that can be detected by depression specific measures. We found only one published study that evaluated the performance of the EQ-5D

For peer review only

in adolescents with depression [34]. This study found a statistically significant correlation between EQ-5D and depression specific outcome measures, though this relationship was stated to be weak. However, there is considerable evidence looking at the use of EQ-5D in adults with depression. A recent systematic review found 14 studies which evaluated the performance of the EQ-5D in patients with depression and/or anxiety [35]. The mean age of participants in these studies ranged from 39 to 49 years of age. The authors of this systematic review concluded that the EQ-5D showed good construct validity and responsiveness for people with depression. These studies provide limited evidence for the use of EQ-5D in adolescents with depression.

Another factor may be that the current study used the 5 level version of the EQ-5D (EQ5D-5L) [27]. All the currently available literature evaluating the EQ-5D in depression uses the 3-level version (EQ5D-3L) [36]. It may be that the 5-level version performs less well in people with depression. An alternative explanation for the small non-significant estimate of the QALY effect of the intervention may simply have been that the sample size was insufficient to detect differences in this generic measure, which we would expect to be less sensitive than the depression specific score. Alternatively, there may be differences in depression related symptoms but these may not be translated into improvements in overall health related quality of life. Further research using and evaluating the EQ-5D-5L in adolescents would be beneficial.

The current study suggests that the intervention can generate improvements in the CDI-2 at approximately £61 per point change in CDI-2 (imputed analysis). This may represent a cost-effective intervention but it is unclear as to what a point change in the CDI-2 should be worth. A study in Korean adolescents suggested that cut of points for mild, moderate, and severe depression on the CDI-2 could be 15, 20, and 25 respectively. [37] A recent meta-



For peer review only - http://bmjopen.bmj.com/site/about/guidelines.xhtml

analysis of utility values of health states related to depression in adults suggested that mild, moderate, and severe depression could be associated with EQ-5D utility values of 0.57, 0.52, and 0.39 respectively. [38] This implies that changes in depression states can have significant implications for EQ-5D, and hence QALYs, and provides some evidence that the CDI-2 differences shown in this study could be meaningful.

Additionally, it is unclear how persistent any benefits generated by the intervention would be. The current study only followed participants up for approximately 6 months. If benefits persisted for more than 6-months they would not be captured by the current design, which therefore may overestimate the cost per point change in CDI-2. Furthermore, the delivery of the intervention in this study was influenced because it was part of a clinical trial. This limited numbers available for each exercise group as some of the potentially eligible participants would have been randomised into the control group. In practice the intervention could be delivered to slightly larger groups with the same resources and hence costs per person may be lower than indicated in this trial.

## Conclusion

Mental health practitioners are committed to using the best available evidence to guide their practice. It is important to demonstrate that any new intervention is cost-effective as well as being clinically effective. There is evidence that exercise can be an effective intervention for adolescents with depression [11] and the current study shows that preferred intensity exercise could also represent a cost-effective intervention in terms of the CDI-2. However, more work would be needed to establish the health economic value of a point change in CDI-2. Incorporating exercise into the repertoire of interventions could add value to the care of children and adolescents receiving treatment and support for depression.

For peer review only

Table 1 Unit costs used in the analysis (UK £2012/13)

| Resource use item | Cost | Source |
|---|---|---|
| Inpatient Stays | Various | NHS reference costs [26] |
| Medicines | Various | British National Formulary [25] |
| Mental health A&E | 228 | NHS reference costs [26] |
| A&E | 115 | NHS reference costs [26] |
| O/P Mental Health | 234 | NHS reference costs [26] |
| O/P Other | 187 | NHS reference costs [26] |
| School Nurse | 14 | PSSRU [23] |
| GP | 37 | PSSRU [23] |
| Paediatrician | 187 | NHS reference costs [26] |
| Physiotherapy | 12 | PSSRU [23] |
| Clinical psychology | 68 | PSSRU [23] |
| Speech therapy | 15 | PSSRU [23] |
| Hearing specialist | 65 | NHS reference costs [26] |
| Other contacts | 28 | Weighted average of reported contacts |
| Counselling/therapist | 59 | PSSRU [23] |
| home help/care worker | 19 | PSSRU [23] |
| Social worker | 51 | PSSRU [23] |
| Overnight stay | 91 | PSSRU [23] |



For peer review only - http://bmjopen.bmj.com/site/about/guidelines.xhtml

Table 2. Descriptive characteristics

| Baseline characteristic | Full data set | | Complete health economics data | |
|---|---|---|---|---|
| | Control | Intervention | Control | Intervention |
| Number | 42 | 44 | 17 | 25 |
| Percentage Female | 81% | 75% | 88% | 88% |
| Percentage White | 98% | 95% | 100% | 92% |
| Age (years) | 15.4 | 15.4 | 15.5 | 15.3 |
| Baseline CDI-2 | 28.2 | 29.1 | 28.7 | 29.4 |
| Baseline EQ5D | 0.82 | 0.78 | 0.82 | 0.74 |
| Time from recruitment to follow-up (weeks) | 39 | 37 | 36 | 36 |

For peer review only

Table 3. Costs at baseline and in the follow-up period for control and intervention groups (£'s)

| | Baseline | | Follow-up | |
|---|---|---|---|---|
| Resource use | Control (mean(SD)) | intervention (mean(SD)) | Control (mean(SD)) | intervention (mean(SD)) |
| Medicines | 8.5 (22.7) | 28.8 (121.1) | 4.3 (11) | 9.5 (21) |
| Inpatient stays | 1142 (4203) | 1046 (4982) | 0 (0) | 44.6 (162.9) |
| Health related A&E | 40.2 (89.6) | 36.5 (85.3) | 40.2 (89.6) | 73 (205.2) |
| Other A&E | 47.4 (81.9) | 27.6 (60.1) | 20.3 (45.2) | 13.8 (50.6) |
| Mental health O/P appointments | 1432 (1892) | 983 (1317) | 771 (1281) | 983 (1525) |
| Other O/P | 44 (140.7) | 29.9 (88.4) | 110 (453.5) | 89.8 (448.8) |
| **Total costs of secondary care** | **2705 (5775)** | **2122 (5570)** | **941 (1310)** | **1204 (1632)** |
| School nurse | 11.3 (21.8) | 71.7 (272) | 22.5 (67.1) | 0 (0) |
| Health visitors | 0 (0) | 0 (0) | 0 (0) | 0 (0) |
| Cost of dental treatment | 10.3 (13.4) | 13.3 (13.9) | 13.9 (18.6) | 8.2 (14.5) |
| Cost of GP visits | 126.2 (88.8) | 126 (175) | 132.8 (107.9) | 72.5 (91.6) |
| Costs of visits to paediatrician | 44 (105.1) | 37.4 (93.5) | 0 (0) | 15 (74.8) |
| Optician visits | 5.9 (9.4) | 8 (12.9) | 1.2 (4.9) | 7.2 (24.4) |
| Physiotherapist | 2.8 (11.6) | 3.8 (16.9) | 0 (0) | 0 (0) |
| Clinical psychology | 0 (0) | 3.6 (18) | 127.1 (358.7) | 0 (0) |
| Speech therapy | 0.9 (3.6) | 0 (0) | 0 (0) | 0 (0) |
| Hearing specialist | 0 (0) | 18.2 (69) | 0 (0) | 2.6 (13) |
| Other visits to health practitioners | 37.5 (59.6) | 13.9 (39.4) | 0 (0) | 0 (0) |
| **Total costs of visits to health practitioners.** | **239 (140)** | **296 (358)** | **297.5 (428.3)** | **105.5 (117.9)** |
| Family counselling | 31.2 (88.8) | 2.4 (11.8) | 0 (0) | 0 (0) |
| Individual Counselling | 226 (282) | 316 (423) | 38.2 (142.9) | 125.1 (589.6) |
| Other Counselling | 76.4 (138) | 14.2 (49) | 13.9 (57.2) | 0 (0) |
| **Total counselling costs** | **333 (375)** | **333 (414)** | **52.1 (150.3)** | **125 (590)** |



For peer review only - http://bmjopen.bmj.com/site/about/guidelines.xhtml

| | | | | |
|---|---|---|---|---|
| Home help care worker | 0 (0) | 6.8 (34.2) | 0 (0) | 15.2 (76) |
| Social worker | 6 (24.7) | 61.2 (250) | 0 (0) | 40.8 (204) |
| After school clubs | 0 (0) | 0 (0) | 0 (0) | 0 (0) |
| Other support services | 1.1 (4.6) | 0 (0) | 0 (0) | 0 (0) |
| **Total social care** | **7.1 (24.9)** | **68 (250)** | **0 (0)** | **56 (280)** |
| Nights in children's home | 0 (0) | 0 (0) | 0 (0) | 0 (0) |
| Nights in foster care | 0 (0) | 0 (0) | 0 (0) | 0 (0) |
| Nights in other residential care | 0 (0) | 364 (1820) | 0 (0) | 0 (0) |
| **Total for residential care** | **0 (0)** | **364 (1820)** | **0 (0)** | **0 (0)** |
| **Illness related service use by family** | **19.9 (64.1)** | **67.6 (137)** | **5.6 (16.2)** | **38.4 (139.8)** |
| **Costs of intervention** | - | - | **0 (0)** | **351 (144)** |
| **Total follow up costs** | **3312 (5980)** | **3280 (6236)** | **1301 (1512)** | **1889 (1853)** |

Based on sample of 42 cases (17 controls and 25 in intervention group).

For peer review only

Table 4 Outcome measures used in the economic analyses at different study time points

| Outcome measure | Control mean (95% CI) | Intervention mean (95% CI) |
|---|---|---|
| Baseline EQ-5D-5L | 0.818 (0.741 , 0.895) | 0.744 (0.683 , 0.804) |
| Post intervention EQ-5D-5L | 0.841 (0.788 , 0.893) | 0.823 (0.761 , 0.885) |
| Follow-up EQ-5D-5L | 0.881 (0.836 , 0.926) | 0.838 (0.768 , 0.908) |
| Time in study | 36.300 (31.4 , 41.3) | 36.300 (32.6 , 40) |
| QALY | 0.594 (0.507 , 0.681) | 0.567 (0.494 , 0.641) |
| Baseline CDI-2 | 28.7 (25.1 , 32.4) | 29.4 (25.2 , 33.6) |
| Follow-up CDI-2 | 24.6 (19.3 ,29.8) | 20.4 (15.9 , 24.8) |



For peer review only - http://bmjopen.bmj.com/site/about/guidelines.xhtml

Table 5. Results of the economic analysis for both cost per point change in CDI-2 and cost per QALY

| Outcome | Analysis | Incremental Cost | CI | Incremental effect | CI | ICER |
|---|---|---|---|---|---|---|
| CDI-2 difference | Complete Case | 334 | (-606 , 1274) | -4.6 | (-10.1 , 0.87) | 73* |
| | Imputed | 292 | (-558 , 1142) | -4.8 | (-10.3 , 0.75) | 61* |
| QALY difference | Complete Case | 355 | (-596 , 1305) | -0.0027 | (-0.04 , 0.03) | Negative |
| | Imputed | 286 | (-1063 , 1634) | -0.0019 | (-0.063 , 0.067) | £152,822 |

*For ICERs related to change in CDI-2 the sign of the difference has been changed as a negative change in CDI-2 represents an improvement

For peer review only

Figure 1 – Cost-Effectiveness Acceptability Curve (CEAC) for cost per point improvement in CDI-2

Figure 2 – Cost-Effectiveness Acceptability Curve (CEAC) for cost per QALY analysis

For peer review only



For peer review only - http://bmjopen.bmj.com/site/about/guidelines.xhtml

The authors declare that they have no competing interests

Authors contributions: PC and TC lead the clinical trial. BG was the trial statistician. TS designed the original economic analysis. DT amended data collection forms and conducted the economic analysis. DT drafted the manuscript. All authors commented on the manuscript and inputted into relevant sections. All authors read and approved the final manuscript. DT takes responsibility for integrity of the health economics analysis reported here.

Acknowledgements

The study was funded by the National Institute of Mental Health, Research for Patient Benefit Programme, grant reference number: PB-PG-1208-18097. The funders played no role in the collection, analysis, and interpretation of data or in the writing of this manuscript.

The authors acknowledge the contribution of all study participants, and their parents, staff of the Child and Adolescent Mental Health Services at Nottinghamshire Healthcare NHS Trust; without whom this study would not have been possible.

For peer review only

## References


1. Thapar A, Collishaw S, Pine DS, et al. Depression in Adolescence. Lancet 2012;**379**(9820):1056-67.
2. Collishaw S, Maughan B, Natarajan L, et al. Trends in adolescent emotional problems in England: a comparison of two national cohorts twenty years apart. Journal of Child Psychology and Psychiatry 2010;**51**(8):885-94.
3. Birmaher B, Brent D, Bernet W, et al. Practice parameter for the assessment and treatment of children and adolescents with depressive disorders. 1503-26 2007;**46**(11):1503-26.
4. Wesselhoeft R, Sorensen MJ, Heiervang ER, et al. Sub threshold depression in children and adolescents - a systematic review. Journal of Affective Disorders 2013;**151**(1):7-22.
5. Babiss LA, Gangwisch JE. Sports participation as a protective factor against depression and suicidal ideation in adolescents as mediated by self-esteem and social support. . journal of Development and Behavioural Pediatrics 2009;**30**(5):376-84.
6. Jerstad SJ, Boutelle KN, Ness KK, et al. Prospective reciprocal relations between physical activity and depression in female adolescents. Journal Of Consulting And Clinical Psychology 2010;**78**(2):268-72.
7. Neissaar I, Raudsepp L. Changes in Physical Activity, Self-Efficacy and Depressive Symptoms in Adolescent Girls. Pediatric Exercise Science 2011;**23**(3):331-43.
8. Sund AM, Larsson B, Wichstrøm L. Role of physical and sedentary activities in the development of depressive symptoms in early adolescence. Social Psychiatry And Psychiatric Epidemiology 2011;**46**(5):431-41.
9. Hughes CW, Barnes S, Barnes C, et al. Depressed Adolescents Treated with Exercise (DATE): A pilot randomized controlled trial to test feasibility and establish preliminary effect sizes. Mental Health and Physical Activity 2013;**6**(2):119-31.
10. Carter T, Callaghan P, Khalil E, et al. The effectiveness of a preferred intensity exercise programme on the mental health outcomes of young people with depression: a sequential mixed methods evaluation. BMC Public Health 2012;**12**(187).
11. Carter T, Guo B, Turner D, et al. Preferred intensity exercise for adolescents receiving treatment for depression: a pragmatic randomised controlled trial BMC Psychiatry 2015;**15**(1):247.
12. Snell T, Knapp M, Healey A, et al. Economic impact of childhood psychiatric disorder on public sector servicess in Britain: estimates from national survey data. Journal of Child Psychology and Psychiatry 2013;**54**(9):977-85.
13. Knapp M, McCrone P, Fombonne E, et al. The Maudsley long-term follow-up of child and adolescent depression: 3. Impact of comorbid conduct disorder on service use and costs in adulthood. British journal of Psychiatry 2002;**180**:19-23.
14. Philipsson A, Duberg A, Mőller M, et al. Cost-utility analysis of a dance intervention for adolescent girls with internalising problems Cost Effectiveness and Resource Allocation 2013;**11**(4).
15. Domino ME, Burns BJ, silva SG, et al. Cost-Effectiveness of Treatments for Adolescent Depression: Results From TADS. American Journal of Psychiatry 2008;**165**(5):588-96.
16. Domino ME, Foster EM, Vitiello B, et al. Relative cost-effectiveness of treatments for adolescent derpession: 36-week results from the TADS randomized trial. Journal of the American Academy of Child and Adolescent Psychiatry 2009;**48**(7):711-20.
17. Wright DR, Haaland WL, Ludman E, et al. The costs and cost-effectiveness of collaborative care for adolescents with depression in primary care settings: A randomized clinical trial. JAMA Pediatrics 2016;**170**(11):1048-54.
18. Byford S, Barrett B, Roberts C, et al. Cost-effectiveness of selective serotonin reuptake inhibitors and routine specialist care with and without cognitive–behavioural therapy in adolescents with major depression. British journal of Psychiatry 2007;**191**(6):521-27.
19. Anderson R, Ukoumunne OC, Sayal K, et al. Cost-effectiveness of classroom-based cognitive behaviour therapy in reducing symptoms of depression in adolescents: a trial-based analysis. Journal of Child Psychology & Psychiatry & Allied Disciplines 2014;**55**(12):1390-7.
20. Stallard P, Phillips R, Montgomery AA, et al. A cluster randomised controlled trial to determine the clinical effectiveness and cost-effectiveness of classroom-based cognitive-behavioural therapy (CBT) in reducing symptoms of depression in high-risk adolescents. Health Technology Assessment (Winchester, England) 2013;**17**(47):vii-xvii, 1-109.
21. Wright B, Tindall L, Littlewood E, et al. Computerised cognitive-behavioural therapy for depression in adolescents: Feasibility results and 4-month outcomes of a UK randomised controlled trial. BMJ Open 2017;**7**(1):e012834.




For peer review only - http://bmjopen.bmj.com/site/about/guidelines.xhtml

22. Kovacs M, Staff. M. *Children's Depression Inventory 2nd Edition (CDI 2) Technical manual.*: Multi-Health Systems, 2011.
23. Curtis L, editor. *Unit Costs of Health and Social Care 2013*. Canterbury: University of Kent, PSSRU, 2013.
24. Beecham J, Knappp M. Costing psychiatric interventions. In: Thornicroft G, ed. Measuring mental health needs. 2nd ed. London: Gaskell, 2001.
25. Joint Formulary Committee. British National Formulary (online). Secondary Joint Formulary Committee. British National Formulary (online) 2014. http://www.medicinescomplete.com.
26. NHS Reference costs. In: Health Do, ed. London: Department of Health, 2013.
27. Herdman M, Gudex C, Lloyd A, et al. Development and preliminary testing of the new five-level version of EQ-5D (EQ-5D-5L). Quality of Life Research 2011;**20**(10):1727-36.
28. Devlin N, Shah K, Feng Y, et al. Valuing Health-Related Quality of Life: An EQ-5D-5L Value Set for England. 2016; 2016(14/10/2016). https://www.ohe.org/publications/valuing-health-related-quality-life-eq-5d-5l-value-set-england.
29. Faria R, Gomes M, Epstein D, et al. A guide to handling missing data in cost-effectiveness analysis conducted within randomised controlled trials. PharmacoEconomics 2014;**32**(12):1157-70.
30. White IR, Royston P, Wood AM. Multiple imputation using chained equations: issues and guidance for practice. Statistics in Medicine 2010;**30**(4):377-99.
31. Manca A, Hawkins N, Sculpher M. Estimating mean QALYs in trial based cost-effectivenss analysis: the importance of controlling for baseline utility. Health Economics 2005;**14**(5):487-96.
32. Marshall A, Altman DG, Holder RL, et al. Combining estimates of interest in prognostic modelling studies after multiple imputation: current practcie and guidelines. BMC Medical Research Methodology 2009;**9**(1):57.
33. Löthgren M, Zethraeus N. Definition, interpretation and calculation of cost-effectiveness acceptability curves. . Health Economics 2000;**9**(7):623-30.
34. Byford S. The validity and responsiveness of the EQ-5D measure of health-related quality of life in aan adolescent population with persistent major depression. Journal of Mental Health 2013;**22**(2):101-10.
35. Brazier J, Connell J, Papaionnou D, et al. A systematic review, psychometric analysis and qualitative assessment of generic preference-based measures of health in mental health populations and the estimation of mapping functions from widely used specific measures. Health Technology Assessment 2014;**18**(34).
36. Brooks R. EuroQol: the current state of play. Health Policy 1996;**37**(1):53-72.
37. Bang YR, Park JH, Kim SH. Cut-off scores of the children's depression inventory for screening and rating severity in Korean adolescents. Psychiatry Investigation 2015;**12**(1):23-28.
38. Kolovos S, Bosmans JE, van Dongen JM, et al. Utility scores for different health states related to depression: individual participant data analysis. Quality of Life Research 2017;**26**:1649-58.

For peer review only

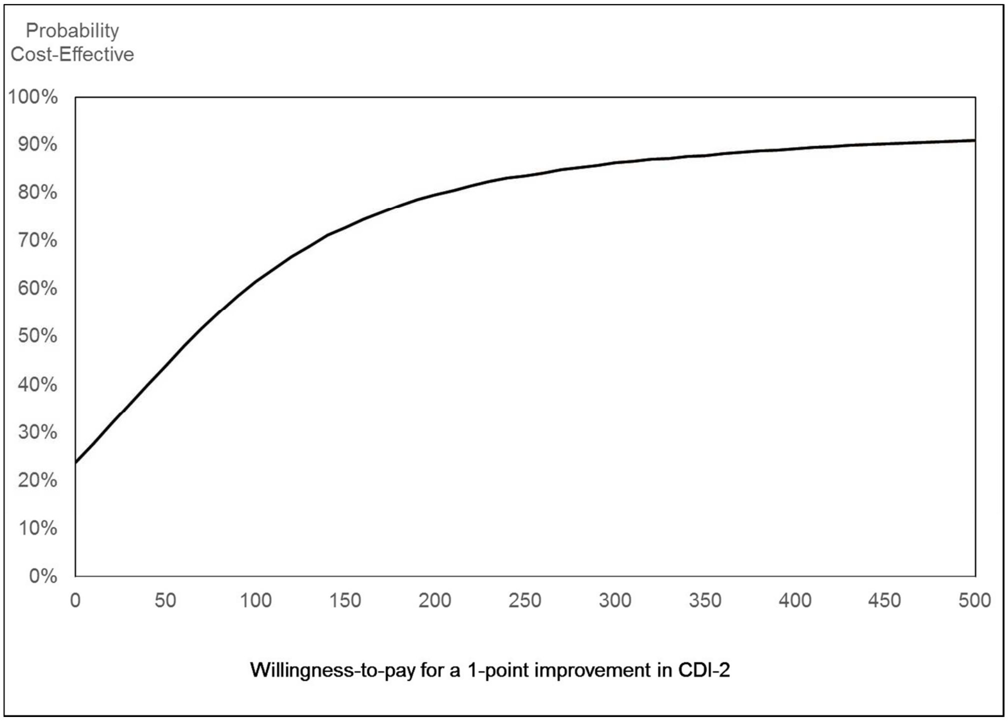


Figure 1 – Cost-Effectiveness Acceptability Curve (CEAC) for cost per point improvement in CDI-2

122x87mm (300 x 300 DPI)

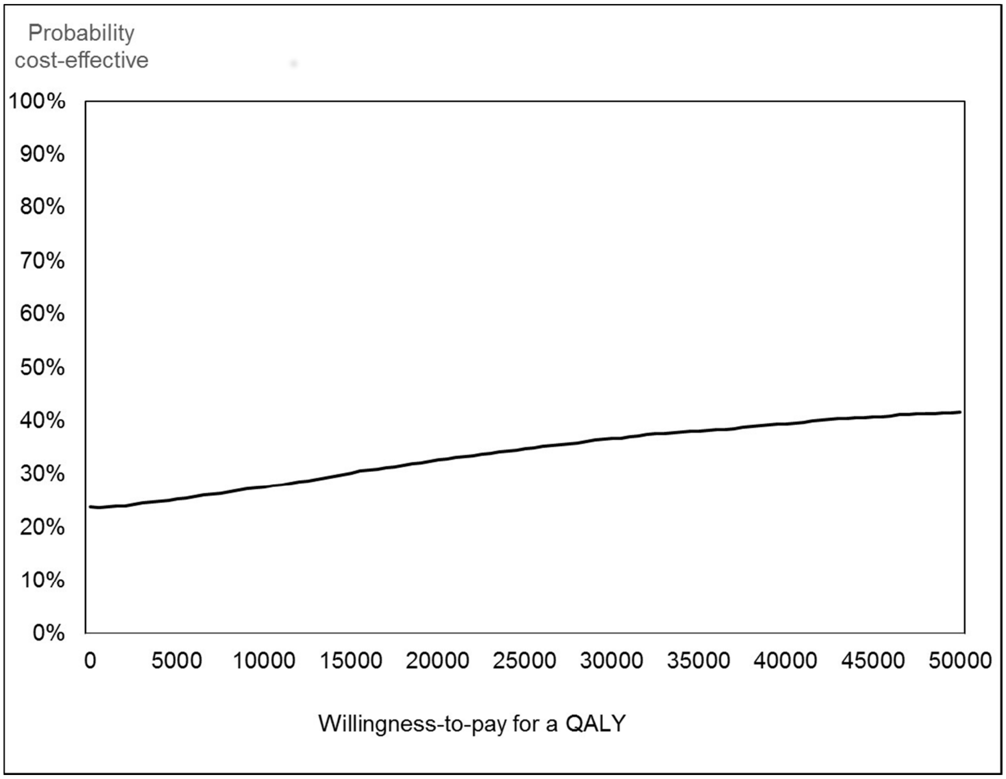


Figure 2 – Cost-Effectiveness Acceptability Curve (CEAC) for cost per QALY analysis

106x82mm (300 x 300 DPI)

For peer review only

CHEERS Statement

| Title and Abstract | Item | Recommendation | Reported on page number/line number |
|---|---|---|---|
| Title | 1 | Identify the study as an economic evaluation or use more specific terms such as 'cost-effectiveness analysis', and describe the interventions compared. | Page 1<br>Specifies cost-effectiveness and names intervention |
| Abstract | 2 | Provide a structured summary of objectives, perspective, setting, methods (including study design and inputs), results (including base case and uncertainty analysis), and conclusions. | Page 2, structured abstract is provided. |
| Introduction | | | |
| Background and objectives | 3 | Provide an explicit statement of the broader context for the study | Yes, page 4-5 |
| | | Present the study question and its relevance for health policy or practice decisions. | Yes, page 4-5 |
| Methods | | | |
| Target population and subgroups | 4 | Describe the characteristics of the base case population and subgroups analysed, including why they were chosen. | Yes, page 5<br>Currently gives details of study population<br>There is no subgroup analysis |
| Setting and location | 5 | State relevant aspects of the system(s) in which the decisions(s) need(s) to be made. | Yes, page 5<br>Tier 2 and Tier 3 CAMHS outpatient services |
| Study perspective | 6 | Describe the perspective of the study and relate this to the costs being evaluated. | Yes, page 6 |
| Comparators | 7 | Describe the interventions or strategies being compared and state why they were chosen. | Page 5-6 |
| Time Horizon | 8 | State the time horizon(s) over which costs and consequences are being evaluated and say why appropriate. | Page 6 |
| Discount rate | 9 | Report the choice of discount rate(s) used for costs and outcomes and say why appropriate. | Page 6 |
| Choice of health outcomes | 10 | Describe what outcomes were used as the measure(s) of benefit in the evaluation and their | Page 8<br>Cost per point change in CDI-2 and cost/QALY |

For peer review only

| | | | |
|---|---|---|---|
| | | relevance for the type of analysis performed | |
| Measurement of effectiveness | 11a | *Single study-based estimates:* Describe fully the design features of the single effectiveness study and why the single study was a sufficient source of clinical effectiveness data. | Page 5-6 – randomised controlled trial section of methods |
| | 11b | *Synthesis-based estimates:* Describe fully the methods used for identification of included studies and synthesis of clinical effectiveness data. | NA |
| Measurement and valuation of preference based outcomes | 12 | If applicable, describe the population and methods used to elicit preferences for outcomes. | NA |
| Estimating resources and costs | 13a | *Single study-based economic evaluation:* Describe approaches used to estimate resource use associated with the alternative interventions. Describe primary or secondary research methods for valuing each resource item in terms of its unit cost. Describe any adjustments made to approximate to opportunity costs. | Cost section Page 6 to 8 |
| | 13b | *Model-based economic evaluation:* Describe approaches and data sources used to estimate resource use associated with model health states. Describe primary or secondary research methods for valuing each resource item in terms of its unit cost. Describe any adjustments made to approximate to opportunity costs. | NA |
| Currency, price date, and conversion | 14 | Report the dates of the estimated resource quantities and unit costs. Describe methods for adjusting estimated unit costs to the year of reported costs if necessary. Describe methods for converting costs. | Page 6 |
| Choice of model | 15 | Describe and give reasons for the specific type of decision-analytical model used. Providing a figure to show model structure is strongly recommended. | NA |
| Assumptions | 16 | Describe all structural or other assumptions underpinning the decision-analytical model. | NA |

For peer review only

| | | | |
|---|---|---|---|
| Analytical methods | 17 | Describe all analytical methods supporting the evaluation. This could include methods for dealing with skewed, missing, or censored data; extrapolation methods; methods for pooling data; approaches to validate or make adjustments (such as half cycle corrections) to a model; and methods for handling population heterogeneity and uncertainty. | Page 9 and 10 – Analysis section |
| Results | | | |
| Study parameters | 18 | Report the values, ranges, references, and, if used, probability distributions for all parameters. Report reasons or sources for distributions used to represent uncertainty where appropriate. Providing a table to show the input values is strongly recommended. | NA |
| Incremental costs and outcomes | 19 | For each intervention, report mean values for the main categories of estimated costs and outcomes of interest, as well as mean differences between the comparator groups. If applicable, report incremental cost-effectiveness ratios. | Table 3 and cost section, page 10. |
| Characterising uncertainty | 20a | *Single study-based economic evaluation:* Describe the effects of sampling uncertainty for the estimated incremental cost and incremental effectiveness parameters, together with the impact of methodological assumptions (such as discount rate, study perspective). | Page 9-12 – results section<br>Table 4<br>Table 5<br>Figure 1<br>Figure 2 |
| | 20b | *Model-based economic evaluation:* Describe the effects on the results of uncertainty for all input parameters, and uncertainty related to the structure of the model and assumptions. | NA |
| Characterising heterogeneity | 21 | If applicable, report differences in costs, outcomes, or cost-effectiveness that can be explained by variations between subgroups of patients with different baseline characteristics or other observed variability in effects that are not reducible by more | NA |

For peer review only

| | | | |
|---|---|---|---|
| | | information. | |
| Discussion | | | |
| Study findings, limitations, generalisability, and current knowledge | 22 | Summarise key study findings and describe how they support the conclusions reached.<br>Discuss limitations and the generalisability of the findings and how the findings fit with current knowledge. | Yes – Page 13<br><br>Yes – Page 13 to 14 |
| **Other** | | | |
| Source of funding | 23 | Describe how the study was funded and the role of the funder in the identification, design, conduct, and reporting of the analysis. Describe other non-monetary sources of support. | Yes – Page 3<br>Role of funders – page 24 |
| Conflicts of interest | 24 | Describe any potential for conflict of interest of study contributors in accordance with journal policy. In the absence of a journal policy, we recommend authors comply with International Committee of Medical Journal Editors recommendations. | Page 24 |

For peer review only
